# Differentiation between early and severe stages of dementia using diagnosis, prescription and utilization patterns

**DOI:** 10.1002/alz70860_100898

**Published:** 2025-12-23

**Authors:** Moritz Platen, Maresa Buchholz, Anika Raedke, Annelie Scharf, Eva Glaeser, Audrey Iskandar, Wolfgang Hoffmann, Bernhard Michalowsky

**Affiliations:** ^1^ Deutsches Zentrum für Neurodegenerative Erkrankungen e. V. (DZNE), site Rostock / Greifswald, Greifswald, Mecklenburg‐Vorpommern, Germany; ^2^ German Center for Neurodegenerative Diseases e.V. (DZNE), Site Rostock/Greifswald, Greifswald, Germany; ^3^ German Center for Neurodegenerative Diseases, Greifswald, Mecklenburg‐West Pomerania, Germany; ^4^ German Center for Neurodegenerative Diseases (DZNE), Greifswald, Mecklenburg–Western Pomerania, Germany; ^5^ Institut for Community Medicine, University Medicine, University of Greifswald, Greifswald, Germany

## Abstract

**Background:**

Routinely collected claims data often lack clinical parameters like dementia severity, which are essential for treatments targeting early disease stages. Diagnoses, prescriptions, and healthcare service utilization patterns are commonly used to infer dementia severity, but evidence validating these patterns is limited.

**Objective:**

To identify and validate predictors (diagnoses, prescriptions, and utilization patterns) for differentiating early and more severe dementia stages.

**Methods:**

This cross‐sectional analysis used baseline data from 737 dementia patients. Comprehensive assessments captured clinical data and healthcare utilization. Diagnoses and prescribed medications were extracted from general practitioner files. Dementia severity was categorized by the Mini‐Mental State Examination (MMSE): early (≥27), mild (20–26), and moderate/severe (0–19). Ordinal logistic regression analyzed associations between predictors and severity, with average marginal effects (AME) quantifying their impact. Specificity and negative predictive values (NPV) were calculated to exclude milder stages.

**Results:**

The sample (56% female, mean age 80) was classified as early (18%), mild (43%), or moderate/severe (39%). Predictors of moderate/severe dementia included differential dementia diagnoses (OR 1.78), antipsychotics (OR 3.22), antidementia drugs (OR 2.12), and having a care level. Conversely, the number of medications (OR 0.91) and therapy use (occupational, speech, or physical; OR 0.68) were associated with milder stages. Antipsychotics reduced early‐stage likelihood by 14% and increased moderate/severe likelihood by 21%. Antidementia drugs reduced early‐stage likelihood by 9% and increased moderate/severe likelihood by 13%. Care level reduced early‐stage probability by 2–16%, while moderate/severe probability increased by 3–34%. Antidementia and antipsychotic prescriptions showed a specificity of 95% and an NPV of 81%, reliably excluding early dementia. For mild dementia, specificity was 94%, but NPV dropped to 52%. Differential diagnoses and care levels had a specificity of 86% and NPV of 81%, effectively distinguishing early dementia.

**Conclusion:**

Prescription and diagnostic patterns reliably distinguish early from severe dementia, validating their use for inferring dementia stages from claims data. Further research should refine these predictors to support guideline‐based, early‐stage dementia therapies.

